# Patient Perspectives on Health Data Privacy and Implications for Adverse Drug Event Documentation and Communication: Qualitative Study

**DOI:** 10.2196/21452

**Published:** 2021-01-20

**Authors:** Serena S Small, Corinne M Hohl, Ellen Balka

**Affiliations:** 1 Centre for Clinical Epidemiology & Evaluation Vancouver Coastal Health Research Institute Vancouver, BC Canada; 2 Department of Emergency Medicine University of British Columbia Vancouver, BC Canada; 3 Emergency Department Vancouver General Hospital Vancouver, BC Canada; 4 School of Communication Simon Fraser University Burnaby, BC Canada

**Keywords:** health information technology, adverse drug events, privacy of patient data

## Abstract

**Background:**

Adverse drug events are unintended and harmful effects of medication use. Using existing information and communication technologies (ICTs) to increase information sharing about adverse drug events may improve patient care but can introduce concerns about data privacy.

**Objective:**

This study aims to examine the views of patients and their caregivers about data protection when using ICTs to communicate adverse drug event information to improve patient safety.

**Methods:**

We conducted an exploratory qualitative study. A total of 4 focus groups were held among patients who had experienced or were at risk of experiencing an adverse drug event, their family members, and their caregivers. We recruited participants through multiple avenues and iteratively analyzed the data using situational analysis.

**Results:**

Of the 47 participants recruited, 28 attended our focus groups. We identified 3 primary themes. First, participants felt that improved information sharing about adverse drug events within their circle of care would likely improve care. Second, participants were concerned about data handling and inappropriate access but believed that the benefits of information sharing outweighed the risks of privacy breaches. Finally, participants were more concerned about data privacy in the context of stigmatized health conditions.

**Conclusions:**

Current conditions for maintaining health data privacy are consistent with participants’ preferences, despite the fact that health data are susceptible to breaches and mismanagement. Information sharing that increases patient safety may justify potential privacy risks. Greater attention to patient concerns and the effect of social and contextual concerns in the design and implementation of health information technologies may increase patient confidence in the privacy of their information.

## Introduction

### Background

Adverse drug events are unintended and harmful outcomes of medication use and a leading cause of emergency department visits and unplanned hospital admissions [1-4]. More than 30% of patients presenting to hospitals with adverse drug events are affected by repeat events that occur because care providers unintentionally re-expose patients to medications that previously caused harm [5]. A lack of effective automated processes to communicate adverse drug event information between health providers and across locations of care contributes to the recurrence of these events.

Poor communication about adverse drug events reflects broader fragmentation and siloed information in health. Recent initiatives at the provincial and federal levels of the government in Canada aim to address these communication gaps. In Ontario, the Digital First for Health Strategy intends to increase the availability of patient records for frontline clinicians and reduce barriers to integration [6]. British Columbia’s Digital Health Strategy seeks to modernize the health system through integration, improved care delivery, and data accessibility for clinicians and patients [7]. This includes increasing access to clinical information through end-to-end medication management using existing clinical information systems [8]. Enabling PharmaNet, British Columbia’s medication dispensing database, to receive and transmit adverse drug event information may support such an undertaking.

### Objectives

Enhanced communication about adverse drug events may improve patient safety but can also reveal sensitive diagnoses to a broader range of clinicians than those currently aware of them. This could introduce privacy concerns for patients, particularly among those living with stigmatized illnesses. We examined patients’ perceptions about the need to share information about adverse drug events to optimize patient safety while maintaining data privacy.

## Methods

### Study Design

We conducted a qualitative study to explore patients’ perceptions of information privacy and sharing in the context of developing software to facilitate adverse drug event documentation and communication. Research ethics boards of the University of British Columbia and Simon Fraser University reviewed and approved the protocol. All participants provided written informed consent.

### Study Setting and Sample

Our target study population included adults (≥19 years) who had lived experience with or were at risk of an adverse drug event and family members and caregivers of patients who had experienced an adverse drug event. All participants lived in the Vancouver area or Whistler, British Columbia, between September 2016 and May 2017. We excluded patients who were living in long-term care facilities, did not manage their own medications, were receiving palliative care, were from out of province, or did not speak English. If we approached a patient who was excluded based on the above criteria, we attempted to recruit a family member or caregiver for participation, if they were present during recruitment.

### Recruitment

We used multiple sampling strategies to recruit patients who had lived experience with an adverse drug event or who were at risk of an adverse drug event because of their age (≥65 years) or exposure to polypharmacy and the family members and caregivers of patients who had experienced an adverse drug event. We recruited in person, through posters, and through web-based advertisements.

From September to November 2016, we recruited those who were experiencing or who were at risk of experiencing an adverse drug event. Emergency department pharmacists recruited a convenience sample of patients presenting to the emergency department of Vancouver General Hospital, a tertiary care hospital in Vancouver, Canada, when completing medication reviews. We placed posters in high-traffic areas in the emergency department and at a hospital-based research center to encourage patients and their family members to contact the research team if they were interested in participating. We also sought to recruit members of the general population by posting web-based advertisements on Kijiji and Craigslist and by snowball sampling from personal connections. In recruitment advertisements, we stated that we were seeking patient opinions on having information about their medication-related problems shared among care providers. Our intent was to reach a broader range of individuals who met our target sample criteria, including those that may not have had direct contact with the acute care setting at the time of the focus groups.

From February to May 2017, we recruited individuals with stigmatized illnesses (HIV and/or substance use disorder) by placing posters at a clinic that provides care to HIV-positive women. We hypothesized that these patients may have specific privacy concerns and also because HIV medications are currently not documented in the provincial medication dispensing database, PharmaNet, in part because of privacy concerns at the time of PharmaNet’s implementation [9].

Among those recruited through snowball sampling from personal connections, there was an established relationship between the researchers and participants before the study. For all others, beyond contact for recruitment purposes, there was no prior established relationship.

### Data Collection

The focus groups followed a semistructured discussion guide developed collaboratively by the research team to address themes relevant to adverse drug event information sharing (Multimedia Appendix 1). The principal investigator (EB) and a research assistant (SS) with expertise in qualitative research created the first draft. Other members of the research team then revised and edited the discussion guide to offer different disciplinary perspectives.

Key themes addressed in the discussion guide were experiences with adverse drug events, knowledge of information-sharing practices, and attitudes about data privacy and privacy policy. We allowed participants to engage in open dialog and ask questions beyond the discussion guide and identify and discuss new concepts that we had not considered.

We held focus groups in a research office at the Vancouver General Hospital. The principal investigator (EB), a female social scientist with extensive experience in qualitative methods, led the focus groups, and a research assistant (SS) attended to take notes. At the beginning of each group, we introduced the researchers present and provided a brief definition of adverse drug events, including examples, providing rationale for the groups. Recruitment materials informed prospective participants that we sought to gather opinions to guide the development of a system to support sharing of information about adverse drug events among health care providers. We reiterated this at the time of the focus groups.

We gathered additional information about the participants, including demographic information, using a short debriefing survey at the conclusion of each focus group (Multimedia Appendix 2). We audio recorded the focus groups, which were then transcribed by a research assistant (SS).

### Data Analysis

We coded and analyzed transcriptions using *NVivo 11* qualitative data analysis software (QSR International, version 11, 2015). We created a provisional coding frame to reflect the thematic structure and discussion guide questions. The structure of the coding frame organized participant comments conceptually along the following themes: data privacy, information sharing, awareness of privacy policy, policy preferences, experience with adverse drug events, and recommendations. We (SS and EB) iteratively coded and analyzed the data using situational analysis, a theoretical and methodological approach that examines contextual, relational, and discursive elements in the data through the concurrent creation of memos and mapping exercises [10].

## Results

### Focus Groups and Participant Characteristics

Of the 47 participants we recruited, 28 attended a focus group. Each focus group had 5 to 8 participants (Table 1). A total of 20 participants (20/28, 71%) were in groups A, B, and C. Of these participants, 65% (13/20) were aged above 65 years and at risk of an adverse drug event, 25% (5/20) were from the general population, and 10% (2/20) were caregivers or family members of patients with adverse drug events. Group D consisted of 8 women (8/28, 29%) recruited from a clinic serving HIV-positive women.

**Table 1:** Focus group composition (N=28).

Group ID	Participants, n (%)
A	8 (29)
B	7 (25)
C	5 (18)
D	8 (29)

Each focus group lasted for 120 min. Most participants (25/28, 89%) completed the debriefing survey (Table 2). Most participants were female and aged above 51 years. Many participants had lived experiences with an adverse drug event, knew someone else who had, or both. Most participants had completed at least some postsecondary education.

**Table 2:** Participants’ characteristics (N=28).

Variable		Participants, n (%)	
**Gender**			
	Male		6 (21)
	Female		19 (68)
	No response		3 (11)
**Age (years)**			
	<20		0 (0)
	20-35		2 (7)
	36-50		5 (18)
	51-65		10 (36)
	>66		8 (29)
	No response		3 (11)
**Experience with adverse drug events**			
	Yes, have lived experienced with an adverse drug event		3 (11)
	Know someone who has experience with an adverse drug event		7 (25)
	Both lived experience and know others who have experienced an adverse drug event		6 (21)
	No, have not experienced an adverse drug event		6 (21)
	Unsure		3 (11)
	No response		3 (11)
**Highest level of education**			
	Some high school		3 (11)
	Completed high school		3 (11)
	Some postsecondary		7 (25)
	Completed college or university		7 (25)
	Some graduate school		2 (7)
	Master’s degree		1 (4)
	Doctoral degree		1 (4)
	No response		4 (14)

### Primary Themes

We identified 3 primary themes about information sharing and privacy in the context of adverse drug event communication. Participant quotes to support each theme are presented in the corresponding textboxes, which are representative of the findings for each theme.

#### Participants Believed Enhanced Information Sharing Among Clinicians Would Improve Care

##### Experiences With Informational Discontinuity of Care

Many participants described experiences with fragmented information sharing (Textbox 1). In some cases, participants experienced negative outcomes as a result of poor information sharing. Participant 1, for example, described how their father-in-law’s experience with poor communication of an adverse drug event affected his long-term health, resulted in unnecessary costs, and emotionally affected the patient and his family. Several participants noted that in the absence of effective information-sharing processes, they took responsibility for information sharing themselves.

**Participant quotes about experiences with informational discontinuity of care.**

- “I don’t think there’s a lot of communication between the doctors. Say you have a GP, you have a rheumatologist, you have an HIV specialist – you tell one doctor one thing and they say, ‘oh I didn’t know that, when did that start?’…So you gotta follow up yourself because he can’t… they get so busy, or they forget, or they don’t care or whatever.” [Participant 25, group D]
- “I have been to [the] emergency department with an adverse reaction twice, and it’s very busy. The doctors who see you don’t have time to write your discharge report up in time for you to take a copy away with you…I would like to have further information and would like to be able to pass it on to my GP.” [Participant 11, group B]
- “I think in my father-in-law’s case, [the lack of information sharing] cost our system more money…there were more doctors involved…there was more angst involved…my father-in-law’s condition plummeted. And there were more people involved, there was more testing done, ambulance was called ten times.” [Participant 1, group A]
- “I don’t think it’s very well shared. If it is, it’s pretty piecemeal.” [Participant 3, group A]
- “I’ve just always heard that adverse effects are supposed to be reported…but I never had any confidence that they were.” [Participant 8, group A]
- “I have learned that in many cases, the people to whom I go for one medical event or another don’t always share the information.” [Participant 12, group B]
- “My family doctor gets everything, but I particularly have to make a point of asking for copies to be sent to a couple of my specialist physicians.” [Participant 11, group B]

##### Benefits of Better Communication

Participants felt that better communication between providers in their circle of care would have a positive effect on health outcomes and could improve their experience with the health system, including improved disease and medication management (Textbox 2). They suggested that communication would lessen the recall burden for patients and their families and that broader information sharing would support clinical decision making, especially in situations in which a patient would be unable to communicate or recall the required information.

**Participant quotes describing the benefits of better communication.**

- “And then…someone who is elderly, who may have dementia, who doesn’t have someone advocating for [them] – that information needs to be shared so that somebody can make sure that they’re making good decisions around their health care and prescription medications.” [Participant 1, group A]
- “[My father-in-law’s] health would have been maintained at a higher level for a longer period of time, had the information been shared more regularly.” [Participant 1, group A]
- “Yes, the more [my care providers] know [about my] medications…the better they’re taking care of me.” [Participant 27, group D]
- “When my mother was admitted and all they need is her care card number…all the information [is] there already, so it’s a lot easier for us.” [Participant 19, group C]
- “You’d think that the more information that your caregivers have…the better off you’re going to be if you have a problem or if you’re unconscious or whatever.” [Participant 20, group C]
- “I think the more we share the information, it’s a huge financial benefit…both the emotional and financial side.” [Participant 7, group A]
- “…We have the language barriers, we have culture barriers, we have all of those things to deal with and it makes things very, very difficult, so another reason for having this information [available] to so many people.” [Participant 3, group A]
- “My view is that I am less concerned about privacy, and more concerned about people [caring] for me having the information that they need.” [Participant 8, group A]

##### Most Participants Preferred Electronic Information Sharing

Many participants supported the use of health information technologies to share adverse drug events and medication information (Textbox 3). Participants viewed electronic communication as quick, easy, and environmentally sustainable while also reducing the risk of lost or misplaced files. Although recognizing these advantages, participants were concerned about data security threats (eg, hackers) and system failure (eg, because of an earthquake). As a result, participants felt that clinicians should not rely exclusively on electronic information sharing and storage. Participants suggested backups to electronic information sharing, including telephone-based communications between clinicians, and electronic or paper-based backups.

**Participant quotes about electronic information sharing.**

- “I personally would want it electronically, just because with the technology age nowadays it’s…the easiest method and the quickest method to transfer information…also it’s more eco-friendly than having all these pieces of paper that might get lost…” [Participant 18, group C]
- “I think I’d want it electronic because it is simpler, and mounds of more paper aren’t necessary…or needed.” [Participant 16, group C]
- “All I’ve gotta say is: is there an app for that?” [Participant 3, group A]
- “I don’t think we should lose the ability to ever…pick up the phone, because there are quick emergency situations that can save a life…but generally I think that electronically is the most practical.” [Participant 7, group A]
- “I’m in favour [of] electronically, but with ensured back up because you could lose everything.” [Participant 8, group A]
- “Digital is my first choice.” [Participant 14, group B]
- “There’s been breaches with confidential medical files when it comes to computers.” [Participant 21, group D]
- “I would want it electronically and [on] paper because…we live in an earthquake zone.” [Participant 17, group C]

#### Participants Were Concerned With Data Handling and Inappropriate Access

##### Participants Believed Professional Roles Should Determine Access Permissions

Participants focused on the different professional groups that would access their health information rather than the information systems that would mediate information sharing (Textbox 4). Role-based access was a recurrent theme, and participants discussed whether access to information was pertinent to every clinical role. For example, group A agreed that pharmacists needed access to patient information but debated whether pharmacy assistants also did. Similarly, participants in groups A and D questioned whether care providers in long-term care facilities (eg, care aides) or allied health professionals (eg, physiotherapists) required full access to medical information or if they had adequate training to manage confidentiality.

**Participant quotes about role-based access.**

- “I would say it would be ok for [my information to be shared] as long as it’s…the doctor... Say…someone on a team…like maybe a social worker of something, they might be valuable on the team, but the medication part would have absolutely nothing to do with them. So, they shouldn’t be having access to that information because they can’t do anything about it.” [Participant 21, group D]
- “When it comes to doctors and nurses…they share. But if…the definition of ‘care team’ is broader than that, then I would need to know who they were and what they were doing [with my information.]” [Participant 17, group C]
- “[Care aides are] usually wonderful people, but they don’t have the information…on how to deal with the ethics of private information. This is my experience with my mother. But yeah, it would depend who it was. I mean my physio doesn’t need to know, right?” [Participant 17, group C]
- “It’s more of a question of what is their education, what is their guidelines…Like a pharmacist…keeps everything confidential. Does the assistant?” [Participant 7, group A]
- “Maybe…the key is who gets the information [is] anybody that has to do with the prescription.” [Participant 3, group A]
- “You run into a whole hornet’s nest when you’re talking about other people getting that information, like for example insurance companies.” [Participant 12, group B]
- “[The doctors] always have [medical] students in their office, right? They come in with the doctors…So the [medical student has] your information, they’ve got everything in that conversation…And where does it go from there?” [Participant 25, group D]
- “I was just going to say that all the medical and allied health, secondary health professions, have confidentiality and privacy as a really major, serious part of their curriculum…It’s as secure as it can be given people.” [Participant 8, group A]

##### Participants Perceived Internal and External Threats to Their Information in Medical Facilities

Participants believed that data stored electronically in medical facilities were unlikely to be secure (Textbox 5). When asked whether they were aware of any breaches of medical information, a small number of participants said yes. At least one breach was mentioned in each group. Several said that they had heard of both clinical and administrative staff mishandling data, including improper disposal, private conversations in public spaces, and inappropriate access of records. Participants had also heard of external threats, including hacking and breaches of Canadian data by American companies; however, none had been firsthand victims of data breaches in medical facilities. Despite this awareness, most agreed that hearing about breaches did not affect their willingness to share their health data. One participant summarized this sentiment by stating that they thought the benefits of information sharing exceeded the risks of privacy breaches.

**Participant quotes about data security.**

- “I think if someone really wanted to get [my medical information] they could get it…People within the office [of] the hospital, if they wanted to get access to it, I think they could get it. Even if they don’t have…official approvals. And, data hacking is advancing.” [Participant 17, group C]
- “I never thought of it as being that confidential…It’s on a computer and in your pharmacy, and there’s a zillion people [that] have access to it. I just accept the system the way it is.” [Participant 20, group C]
- “[Information is] only as secure as the individuals handling it.” [Participant 3, group A]
- “I don’t think pharmacies are very secure.” [Participant 15, group B]
- “There’s a lot of hackers out there that can get access to [your information].” [Participant 25, group D]
- “There’s faxes that have…gone to the wrong fax number, so there’s a breach of confidentiality there.” [Participant 21, group D]
- “And where the breaches occur are chatting down the hallway, nurses chatting in the elevator, people in the cafeteria…” [Participant 8, group A]
- “I’ve heard of…an operator throwing some CDs or…storage device in the garbage and then somebody went in the garbage and pulled it out, and there’s half a million records on there.” [Participant 3, group B]
- “I mean, there have been serious privacy breaches on record with the provincial government specifically.” [Participant 15, group B]
- “A whole stack of personal information and somebody just dumped it out in the back lane somewhere.” [Participant 14, group B]

#### Privacy Concerns Are Amplified When Considering Stigma and Potential Discrimination

Discussions about stigma and discrimination around health data emerged in most groups (Textbox 6). Many felt that sharing information about stigmatized illnesses, which could occur if an adverse drug event to HIV medication was recorded, should occur only within a patient’s circle of care, which is consistent with current data privacy standards. Among participants in group D (who live with stigmatized illnesses), concerns about the effects of stigma and discrimination were amplified. Previous experience with the health system that had reduced complex lived experiences with negative labels colored this group’s perception of the system. There was a relationship between trust, willingness to share health information, and stigma. One participant, for example, commented that they might withhold medical information if they did not trust their care provider or had concerns about where their information was going and who could access it. Although we sought to understand information-sharing preferences in clinical settings, several participants mentioned unprompted that their sensitive medical information should not be shared with colleagues or employers. Participants suggested ways to reduce stigma, including educating clinicians and providing information for patients during care encounters.

**Participant quotes about stigma and discrimination.**

- “When [a care provider] comes in and [is] talking about someone’s health, you just don’t outright say ‘hey how’d you get that?’ That’s really disrespectful to a person whether they have diabetes, cancer, HIV, or Hep C, or whatever their medical situation is.” [Participant 21, group D]
- “So, depending on who’s accessing that information…that’s where stigma, discrimination comes in, because [the patient] could…end up being judged from the medication they’re on because [the care provider] knows what those medications are used for.” [Participant 21, group D]
- “I remember all the stigma around cancer when I was a child. It was like the ‘c-word’. You didn’t even call it cancer. And it’s great to see the shift now…It would be lovely if we could get that way with stuff like mental health, Hep C, HIV. You know, there’s definitely more awareness out there but unfortunately there’s…the ignorance.” [Participant 21, group D]
- “I’ll tell you right now, the honest truth about the Downtown Eastside is [care providers] don’t care about you. You’re just a number, you’re just an addict, you’re just a prostitute, you’re just a drunk…You’re not a human being…You’re just shuffled through, seen by whoever’s there [at the clinic] …They don’t [have] your files.” [Participant 28, group D]
- “There was the case of the woman refused entry into the US because she was on anti-depressants.” [Participant 17, group C]
- “I have a mental illness…if I’m a danger to myself or to others, and I’m not taking my meds…then yes, my diagnosis along with my meds need to be passed on to somebody…if I’m stable, then just my medication [information should be shared].” [Participant 4, group A]
- “You know, [doctors] want you to tell them everything…but I don’t want to tell you [doctors] this part, because I don’t trust you guys.” [Participant 25, group D]
- “I just feel…the students…or the…residents, they need to be better educated on…bedside manners.” [Participant 21, group D]
- “I do see more often that…the confidentiality blurbs are out there more often when you’re signing things, [saying] this is how we protect information. That never used to be out there, so I think there is more awareness out there, but I think it needs to continue, like even…ramp it up. And not [allow] people [to] get…complacent about it.” [Participant 21, group D]

## Discussion

### Principal Findings

We explored patients’ perceptions about information privacy and sharing in the context of developing a health information technology that will enable electronic documentation and automated communication of adverse drug event information between providers and across care settings. Most participants supported improved information sharing about adverse drug events, expected technologies, and clinicians to protect their privacy and understood that a lack of information sharing could pose a greater risk to their safety than potential threats to privacy. In the following sections, we explore how existing organizational and institutional measures to protect data privacy are consistent with participant expectations.

Privacy legislation provides a framework for data management. In British Columbia, the Freedom of Information and Protection of Privacy Act (FIPPA) governs the collection, use, and dissemination of personal information by public entities, including health authorities and hospitals. Under FIPPA, public entities must enact and enforce security measures to prevent unauthorized collection, use, access, disclosure, and disposal of personal information. Public entities such as hospitals and clinics must notify individuals that their information is being collected and used but do not require patient consent to share patient information with other members of the care team [11]. Our study participants’ privacy preferences were consistent with these legislative requirements: participants were favorable toward information sharing among clinicians but were wary of giving access to those outside their circle of care (eg, an insurance company) or among those within the circle of care for whom information about an adverse drug event is irrelevant to their role (eg, a physiotherapist). A system that supports adverse drug event data sharing among a patient’s circle of care must minimize the barriers to effective communication and should not require additional patient consent.

Improvement in communication about adverse drug events may be achieved by leveraging existing health information technologies, such as PharmaNet. PharmaNet employs numerous data management safeguards, including physical security (eg, limited access to equipment), operating system security (eg, user access keys), network security (eg, firewall), and screen security (eg, only certain items viewable based on each user’s security profile) [12]. It also adheres to the principles of role-based access, wherein different user groups have different access permissions, which was strongly preferred among participants. In addition, individuals can find out when their record is accessed and those with further privacy concerns can add a password to their PharmaNet profile, allowing them to determine who can or cannot view their profile. This is valuable for individuals living with stigmatized illnesses.

Despite the privacy measures implemented in health information technologies, security under real-world conditions is more volatile. Breaches of health data have been a recurrent focus of media attention and critique, including incidents involving PharmaNet. In 2014, for example, approximately 1600 profiles were compromised by an unknown, unauthorized individual using a doctor’s account [13]. In 2017, more than 20,000 profiles were breached [14]. These breaches exemplify our participants’ concerns and demonstrate the challenge of managing the risks associated with privacy breaches while ensuring that data are accessible in the interest of patient care. Following these events and other privacy concerns, the provincial Ministry of Health introduced a new project to support user management for PharmaNet, which will streamline access approval when implemented [15].

In addition to legislative frameworks, concerns about handling sensitive health information can be addressed in the design of systems by implementing role-based access functionality, building complex password requirements, and regularly auditing use and users. Participants’ concerns regarding threats to data security among staff in medical facilities can be addressed through other nontechnical approaches. Implementation should incorporate education that addresses safe information handling, including proper methods of sharing data and disposing of paper records, and strategies for maintaining the security of log-in credentials. These measures may increase clinicians’ ability to maintain the security of information in their custody while increasing patients’ confidence in the privacy of their information and in the efficacy of information sharing in health.

### Limitations

Sample composition is the primary limitation of this study. Participant self-selection and recruitment from an urban area may have introduced selection bias. More women participated than men. Participants in rural regions, men, and those with other health trajectories or access points within the health system may have different experiences in the health sector that are not reflected. As such, our findings may not translate to other regions, populations, and health conditions. In addition, we did not screen participants who volunteered via classified websites (n=5) to determine whether they met the defined sample criteria (ie, at risk of or experienced an adverse drug event or a family member or caregiver). Therefore, we cannot verify whether all responses are representative of these sample criteria.

### Conclusions

Participants were generally supportive of enhanced informational continuity of care about adverse drug events to facilitate care delivery. The belief that enhanced information sharing would improve care and that a lack of information sharing poses safety risks indicates patient support for broader use of information and communication technologies (ICTs) in health. Privacy considerations were important to participants but largely in the context of the human actors handling the data rather than the electronic systems that mediate information transfer. Fears about stigma and discrimination were prominent drivers, particularly among patients who had experienced stigmatization. Our findings suggest the need to consider the ways that social and contextual factors (eg, living with a stigmatized illness) that affect patient privacy can be addressed at both the human and technical levels in the design and implementation of ICTs in health.

## Appendix

Multimedia Appendix 1 Discussion guide.

Multimedia Appendix 2 Follow-up survey.

## Abbreviations

FIPPA: Freedom of Information and Protection of Privacy Act
ICT: information and communication technology
