# Supplementary material for: Patient Perspectives on Health Data Privacy and Implications for Adverse Drug Event Documentation and Communication: Qualitative Study
Source: J Med Internet Res. 2021 Jan 20;23(1):e21452. doi: 10.2196/21452 (PMC7857938; doi:10.2196/21452)
Supplement: Multimedia Appendix 1 [file jmir_v23i1e21452_app1.docx]

**Patient perspectives on health data privacy and implications for adverse drug event documentation and communication: A qualitative study**

**APPENDIX A: DISCUSSION GUIDE FOR FOCUS GROUPS**

A) Questions about baseline perceptions

1. If you had a problem with a drug and your doctor knew about it, do you think that information is shared with other health care providers? Tell us what you think happens.
2. Is it the same for all providers? (e.g., your family doctor vs. the hospital vs. a specialist?)
3. Do you think there are differences in terms of type of information, in terms of what is and is not shared? Can you explain?

B) Experience with ADEs

1. Have you or someone you know ever experienced an adverse drug event?
   1. [*If yes*] Did you seek medical attention during or following this event? Where did you go? (e.g., family physician, emergency room, etc.)
   2. [*If yes*] Are you aware of whether your care provider documented that event at all? *Probe for further details.*
      1. Do you know if they reported that event to Health Canada’s voluntary adverse drug reaction program? [*show reporting form]*
2. Health Canada’s adverse drug reaction program has the option for patients to report their own reactions. Is self-reporting something that you would engage in if you were to experience an adverse drug event in the future?
   1. Why / Why not
   2. What do you think would make you more likely to self-report?
3. Thinking about reporting or documenting ADEs, who would you want to know about the event? Why?
4. Thinking about an ADE that either you or someone you know experienced, can you give any examples about how better information sharing might have led to a better end results?

C) Attitudes toward sharing medication information

1. Is there anyone you do not want to know about the medications you take? If so, who?
2. How comfortable are you with your care providers sharing your confidential medication information with each other?
   1. [*Based on responses, probe for details related to support for medication info sharing or opposition toward medication info sharing. What are the main drivers for support / opposition?]*
   2. Is there specific information related to your medications you would like shared? Why?
   3. Is there specific information related to your medications you would not want to be shared? Why?
3. Is there anything that would make you more comfortable with the sharing of this type of information? If so, what?
4. Are you comfortable having the provincial government determine what policies should be in place for sharing of medication information?
   1. If not, why not? What do you think they won’t do well? What do they do well?
   2. Is your opinion about policy setting around medication sharing the same or different for the federal government? Why or why not?
5. In British Columbia, five health authorities are responsible for the health delivery and planning in their respective geographic areas. Here at VGH, we are part of the Vancouver Coastal Health Authority. Are you comfortable having the health authorities determine the policies related to medication information sharing?

a. If not, why not? What do you think they won’t do well?

1. If you think that information should be shared, which methods would you prefer – on paper or electronically? Why?
2. Is there anything that could be done to make you more comfortable with sharing information electronically? If so, what?
3. Do you think you should be asked permission each time information about your medications is shared or used by care providers? (For example, when a doctor in the hospital faxes a discharge summary to your family doctor or pharmacist)
   1. What about for research purposes? (For example, when you complete a survey about your health status)
   2. What about information about you that would be used for care and research purposes? (For example, if you break your arm and arrive in hospital, and they record information about the incident explicitly for research purposes, and then that information is sent to a secure computer outside of the hospital)?
   3. What about if the information collected for research purposes was information about pharmaceutical use?
4. Are there any other specific issues you are concerned about regarding sharing of patient information?

C) Attitudes toward data privacy

1. How secure do you think your confidential medical information is in health care facilities, like pharmacies, hospitals, and doctors’ offices?
2. Do you think that removing specific, identifiable information about you will protect your data?
3. What laws in Canada are you aware of, if any, that protect your confidential medical information?

[*Write down responses; fill in gaps as required, provide handout on privacy laws and Vanessa’s law*]

- 1. Do you think there’s a need for stronger legislation surrounding the protection of confidential medical information? What would that look like?
  2. Given the information about different kinds of privacy legislation and the provisions in Vanessa’s Law that require information about adverse drug events to be sent to Health Canada, how secure do you think your confidential medical information is with the government?
  3. Do you think the laws governing privacy interfere with the provision of quality care?
  4. From your perspective, what would be ideal in terms of allowing the sharing of information for health care?

1. Have you heard of any breaches of confidential medical data in the news recently?
   1. [If yes] – What did you hear?
   2. Did this change how you felt about sharing your data with care providers or the government?
   3. [If no] do you think hearing about these types of breaches would have an impact on your willingness to share your medical data with care providers or the government? Why or why not?

D) Concluding / wrap up question: How important do you think data sharing is in relation to data privacy? In other words, if privacy could not be guaranteed, would you still be willing to share medical data?
