# Supplementary material for: Patient Perspectives on Health Data Privacy and Implications for Adverse Drug Event Documentation and Communication: Qualitative Study
Source: J Med Internet Res. 2021 Jan 20;23(1):e21452. doi: 10.2196/21452 (PMC7857938; doi:10.2196/21452)
Supplement: Multimedia Appendix 2 [file jmir_v23i1e21452_app2.docx]

Thank you for participating in our project! To conclude this session, we have a couple of questions we hope you will take the time to answer:

1. Have you or someone you know ever experienced an adverse drug event? (Check all that apply)

- - YES, I have experienced an adverse drug event
  - YES, someone I know has experienced an adverse drug event
    - If YES, what is your relationship to this person (friend, parent, etc.):
  - NO, I have not experienced an adverse drug event, nor has anyone I know
  - I’m not sure

1a. [If YES to the above] In your own words, what was the outcome of this event? If you would like, please describe the event in as much or as little detail as you wish. If you need more room, please feel free to use the opposite side of the sheet.

3. What is your age (in years)?

4. What is your sex?

5. What is the highest level of education you have completed?

- - Some high school
  - Completed high school
  - Some college or university
  - Completed college or university
  - Some graduate school
  - Master’s degree
  - Doctoral degree
  - Legal or medical degree

6. How did you hear about this project?

- - From a researcher in the Emergency Department at VGH
  - From posters at VGH
  - Online (Craiglist, Kijiji)
  - Through our website (<http://actionade.org>)
  - Word of mouth
  - Other (please explain)___________________________________________________

Thank you again for participating!
